# Acute Toxicity and Pharmacokinetic Profile of an EU-GMP-Certified *Cannabis sativa* L. in Rodents

**DOI:** 10.3390/ph16050694

**Published:** 2023-05-03

**Authors:** Leontina-Elena Filipiuc, Raluca Ştefănescu, Carmen Solcan, Mitică Ciorpac, Andrei Szilagyi, Dana Cojocaru, Gabriela Dumitrita Stanciu, Ioana Creangă, Cătălin-Cezar Caratașu, Daniela-Carmen Ababei, Roxana-Elena Gavrila, Andrei-Daniel Timofte, Silviu-Iulian Filipiuc, Veronica Bild

**Affiliations:** 1 Advanced Research and Development Center for Experimental Medicine (CEMEX), Grigore T. Popa University of Medicine and Pharmacy, 16 Universitatii Street, 700115 Iasi, Romania; 2 Faculty of Veterinary Medicine, Ion Ionescu de la Brad University of Life Sciences, 700490 Iasi, Romania; 3 Pharmacodynamics and Clinical Pharmacy Department, Grigore T. Popa University of Medicine and Pharmacy, 16 Universitatii Street, 700115 Iasi, Romania; 4 Histology Department, Grigore T. Popa University of Medicine and Pharmacy, 16 Universitatii Street, 700115 Iasi, Romania

**Keywords:** *Cannabis sativa* L., EU-GMP, delta-9-tetrahydrocannabinol, cannabidiol, acute oral toxicity, pharmacokinetic profile, LD50, rats

## Abstract

The conundrum of *Cannabis sativa*’s applications for therapeutical purposes is set apart by the hundreds of known and commercially available strains, the social, cultural and historical context, and the legalization of its use for medical purposes in various jurisdictions around the globe. In an era where targeted therapies are continuously being developed and have become the norm, it is imperative to conduct standardized, controlled studies on strains currently cultivated under Good Manufacturing Practices (GMP) certification, a standard that guarantees the quality requirements for modern medical and therapeutic use. Thus, the aim of our study is to evaluate the acute toxicity of a 15.6% THC: <1% CBD, EU-GMP certified, *Cannabis sativa* L. in rodents, following the OECD acute oral toxicity guidelines, and to provide an overview of its pharmacokinetic profile. Groups of healthy female Sprague-Dawley rats were treated orally with a stepwise incremental dose, each step using three animals. The absence or presence of plant-induced mortality in rats dosed at one step determined the next step. For the EU GMP-certified *Cannabis sativa* L. investigated, we determined an oral LD50 value of over 5000 mg/kg in rats and a human equivalent oral dose of ≈806.45 mg/kg. Additionally, no significant clinical signs of toxicity or gross pathological findings were observed. According to our data, the toxicology, safety and pharmacokinetic profile of the tested EU-GMP-certified *Cannabis sativa* L. support further investigations through efficacy and chronic toxicity studies in preparation for potential future clinical applications and especially for the treatment of chronic pain.

## 1. Introduction

Cannabis, a plant of the *Cannabaceae* family, has been cultivated and used for millennia for various medical/therapeutical, religious [1], recreational and textile purposes [2,3]. In 1850, due to its supposed medicinal benefits, cannabis was recognized as an official, licit drug and registered in the US Pharmacopeia. The recreational use of cannabis in the US grew steadily from the 1930s until the 1970s, when the Controlled Substances Act categorized it as a Schedule I illicit drug, the most tightly restricted category, making its possession a federal crime. In 2012, the legalization of its ‘recreational’ use in some states in the US and Uruguay fueled a debate about the laws prohibiting or allowing the consumption and supply of cannabis worldwide.

In Europe, a limited distribution system has been registered in the Netherlands since the 1970s, with further developments arising in recent years. The advantages and disadvantages of this regulated system were closely monitored [4]. International plans to legalize cannabis plants have generated concerns that it could lead to an expansion of use and its associated harm, as well as questions about the ways in which the non-medical use of cannabis might be regulated to allay these worries [5].

Recent studies estimate that in 2019, in the US, over 48.2 million people aged 12 and over used cannabis at least once. Also, in Europe, cannabis appears to be the most commonly consumed illicit drug, and it has been estimated that at least one in eight young adults used cannabis in the past year [6,7]. In 2022, medical cannabis has been legalized in 38 states of the US, while recreational use is allowed in 19 other countries [8]. International legislation does not prevent the use of cannabis as a medicine to treat specific pathologies. According to the United Nations framework conventions, the drugs regulated under the international control system should be limited to “medical and scientific purposes”. In some European countries, licensed medicines may contain tetrahydrocannabinol in capsules, dried cannabis flowers for tea or vaporization and cannabis extract as a mouth spray [4].

Since the isolation of the first components of the cannabis plant, cannabidiol (CBD, Adams and Hunt in 1940) and tetrahydrocannabinol (THC, Goani and Mecholum in 1964), more than 500 phytochemicals have been defined to date, of which about 100 are cannabinoids [9,10,11]. For medicinal cannabis strains, the Netherlands Office of Medicinal Cannabis allows for varying levels of THC and CBD, ranging from less than 1% to over 22% and less than 1% to about 9%, respectively. These concentrations are influenced by the cannabis strain, soil, climate, and cultivation techniques. Moreover, the amount absorbed by the body depends on the way the administration route. The use of cannabis for therapeutic and recreational purposes comes with several reported side effects such as hallucinations, variations in blood pressure, dizziness or drowsiness, increases in heart rate, nausea and confusion. Additionally, some big question marks remain about the more severe side effects, such as stroke and/or heart attack. These effects vary with dose, administration route, previous experience and/or the expectations of the user, and the social environment [12,13].

The various pharmacological effects of cannabis do not seem to be limited to psychological properties, having the ability to respond to needs such as the relief of chemotherapy-derived nausea, vomiting, and anorexia [14]; spasticity associated with multiple sclerosis [15,16]; symptomatic mitigation of neurodegenerative disorders [17,18] or HIV/AIDS-related neuropathy and poor appetite [19]. Cannabis has a long tradition as an analgesic and alleviator of neurological symptoms. The cannabinoid receptors (CB1 and CB2) are widely expressed in the central nervous system (CNS) [20]. The activation of the CB1 and CB2 receptors in the presynaptic terminal is associated with the inhibition of voltage-gated Ca channels and the cAMP/PKA pathway, which decreases the release of neurotransmitters and thus inhibits the perception of pain Pl [21]. Multiple receptors, enzymes, and ion channels, including TRP ankyrin type 1 (TRPA1), the vanilloid receptors types 1 and 2 (TRPV1 and TRPV2), TRP melastatin type 8 (TRPM8) [22], and the cyclooxygenase (COX) enzyme have been identified as the targets of an array of compounds isolated from *Cannabis* [23].

With hundreds of known and commercially available cannabis strains [24], from numerous suppliers, with variable standards of growing, production, and manufacture with inconsistent quality controls, pesticide or contaminants levels, and varying genetics, THC, CBD, or terpene profiles, we need to deepen the knowledge about the toxicity and pharmacokinetic profile of cannabis strains cultivated under the Good Manufacturing Practices (GMP) certification. This is a standard that guarantees pharmaceutical-grade quality control and compliance for the correct, constant, and reproducible medical and therapeutic use of cannabis plants.

The current toxicity studies date from the 1970′s and involved cannabis strains [25,26,27] that were grown amateurly outdoor, and therefore cannot be reliably referred to when compared to the high-quality, indoor-grown, tightly-controlled, single-clone, GMP-level products now available on the market. Consequently, modern, up-to-date toxicity studies are needed on these pharmaceutical-grade plants, manufactured under GMP certification, so that the tested products can be used safely in subsequent preclinical and clinical studies. The published results on the use of cannabis for medical purposes in various pathologies can be considered insufficient and, most of the time, are inconclusive because of the heterogeneity of these mostly observational, retrospective studies conducted on various plant sources, suppliers, or manufacturing standards, variable formulations (capsules, tablets, baked products, oils, or decoctions) with different concentrations from various suppliers or manufacturing standards, and thus are unlikely to be reproducible [28,29]. The most studied oral forms are capsules and tablets, but the existing studies reflect a high variability regarding the absorption of cannabinoids from oral forms with different plasma concentrations. A recent systematic review concluded that THC administered orally has a variable pharmacokinetic profile, which differs between formulations, with high variability, including in the case of similar formulations [30]. Furthermore, data on the demographic-specific impact of cannabis use [29,31,32,33], substance use disorders [34,35,36], abuse, dependence and risks come only from retrospective reports [37,38]. Therefore, randomized controlled trials on standardized GMP-level cannabis products are needed to reduce the results’ unpredictability when using medical cannabis, to increase the efficacy and safety of compound formulations for therapeutic uses, and to support further investigations regarding the use of cannabis in specific conditions and pathologies. The data from preclinical toxicity studies on GMP-level cannabis are the first step in this direction and will prove invaluable for modern-day physicians when prescribing or recommending cannabis products or extracts more safely, even though still based on evidence of traditional or current practice use.

In this evolving and dynamic landscape, the objective of our study is to evaluate the acute oral toxicity, LD50 and the pharmacokinetic profile of an EU GMP-certified batch of a dried inflorescence of *Cannabis sativa* L. with 15.6% THC and <1% CBD, following the OECD 423: acute oral toxicity guidelines. Our data open new perspectives in understanding the mechanisms of acute toxicity and clarify the possible health hazards and risk assessment compulsory for clinical trials.

## 2. Results

### 2.1. Acute Oral Toxicity Assessment in Rats

#### 2.1.1. Primary Monitoring and General Health Status

**Figure 1:**
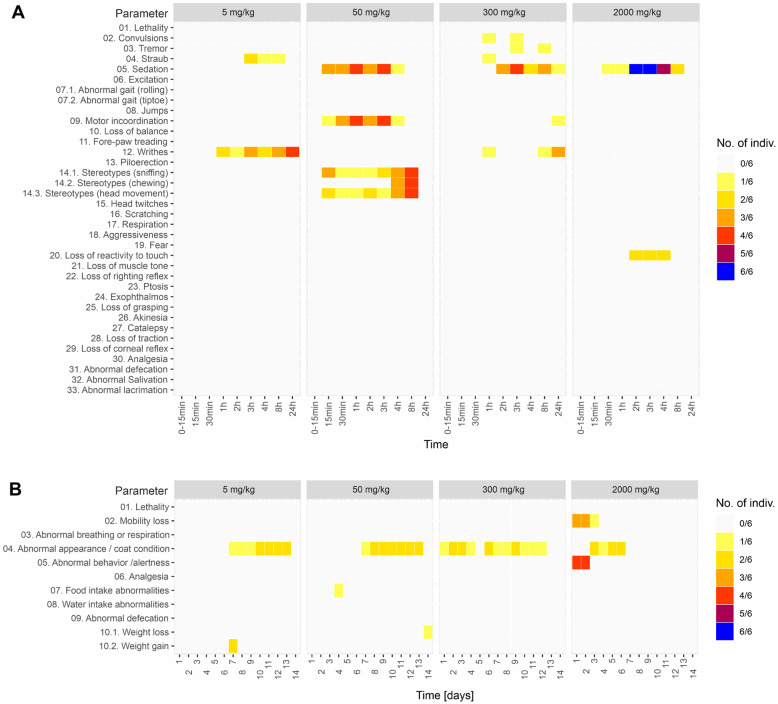
(**A**) Primary monitoring parameters in the first 24 h after oral administration of EU-GMP certified *Cannabis sativa* L. (**B**) General health parameters monitored for 14 days after the oral administration of EU-GMP certified *Cannabis sativa* L.

Monitoring in the first 24 h revealed the following effects (Figure 1A):

- Writhing for the 1–24 h interval from administration for the dose group 5 mg/kg and the dose group 300 mg/kg;
- Straub phenomena in the 1–8 h interval, for the dose group 5 mg/kg and the dose group 300 mg/kg;
- Moderate sedation, maintained for 24 h, for the dose group 50 mg/kg, 300 mg kg and 2000 mg/kg;
- Mild motor incoordination for the 50 and 300 mg/kg dose groups. This effect occurred in the first 8 h from administration for the 50 mg/kg dose group and later, after 12–15 h for the 300 mg/kg dose group;
- Stereotypes (head movements, chewing and sniffing) occurred exclusively for the 50 mg/kg group in the first 8 h;
- Short-duration facial tremors and abortive seizures during the initial 8 h, limited to 16% of the subjects, for the 300 mg/kg group;
- Loss of reactivity to touch during the first 8 h for 16% of the subjects, for the 2000 mg/kg group.

Overall, the health status was unaffected by oral administration of *Cannabis sativa* L. for doses up to 300 mg/kg (Figure 1B). Also, no significant deviation from the normal could be observed for general behavior, the alertness state, food or water intake, and body weight variation (>10%) for the first three doses (5, 50, and 300 mg/kg). The only change over the 14 days of monitoring was the appearance of the rough coat with various frequencies. The oral administration of *Cannabis sativa* L. at 2000 mg/kg dose induced a mobility reduction and a low attention state in the first two days, while the rough coat or porphyrin staining was exhibited from the 3rd to the 6th day. Porphyrin staining (salmon-pink/rust-colored/orange-red) is a normal secretion secreted by the Harderian glands of rats, and a small amount can occasionally be seen near the eyes or nostrils and on the fur.

#### 2.1.2. Paraclinical Evaluation: Gross Necropsy, Biochemistry and Histopathology

At 14 days after oral administration of the EU-GMP certified 15.6% THC <1% CBD *Cannabis sativa* L., the external examination of the animals at necropsy did not reveal any abnormalities in the mouth, nostrils, anus, coat and limbs. The rats had a body condition score of 3 (segmentation of vertebral column slightly palpable, moderate subcutaneous fat deposit over the pelvis, a moderate fat store around the tail base, and the caudal vertebrae were palpable but not segmented). No lesions or signs of disease were observed during internal examination.

The serum biochemical parameters resulting from the oral administration of the compound, graded as the mean of the group +/− SEM for each of the tested doses, are presented in Table 1. The standardized biochemical panel was thus chosen for the indirect quantification of liver and kidney function, the main organs responsible for studying the pharmacokinetics of cannabinoid compounds. The biochemical tests revealed mild alterations in serum creatinine values but without statistical significance when compared to the control group. For the biochemical parameters representative of the liver function, decreases in some aspartate aminotransferase (AST) values were observed at the highest dose (2000 mg/kg), as well as an increase in alkaline phosphatase (ALP) values, but still without statistical significance for either one of these parameters, at none of the tested doses.

Serum glucose levels were slightly below the reference limits in the groups that received low doses of the compound, at 5 mg/kg, respectively at 50 mg/kg, these values increasing and returning to the reference range with the increase in the administered dose. For the rest of the serum biochemistry parameters, no significant alterations between the control and the experimental groups could be observed.

**Figure 2:**
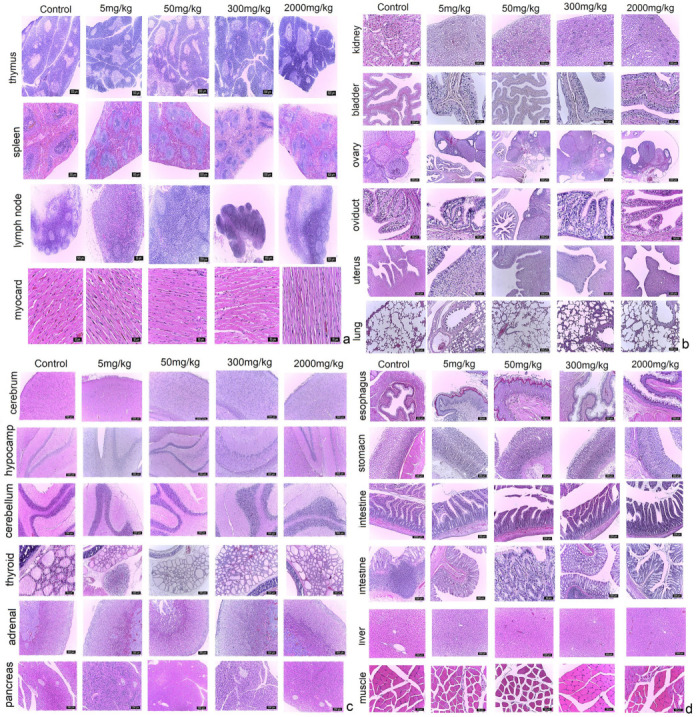
(**a**) Histological aspects of the thymus, spleen, lymph node and myocardium in rats exposed to various doses of EU-GMP certified *Cannabis sativa* L. In the image, it can be seen that no histological changes were recorded, hematoxylin and eosin (H&E) standard staining protocol; (**b**) Morpho-structure of the kidney, urinary bladder, ovary, oviduct, uterus and lung in rats exposed to various doses of EU-GMP certified *Cannabis sativa* L. The organs studied did not show histological changes regardless of the dose; H&E coloration; (**c**) Morpho-structure of the cerebral cortex, hippocampus, cerebellar cortex, thyroid, adrenal gland and pancreas in rats exposed to various doses of EU-GMP certified *Cannabis sativa* L. We mention that no histological changes occurred regardless of the dose, H&E coloration; (**d**) Histological aspects of the esophagus, stomach, small intestine, large intestine, liver and skeletal muscles in rats exposed to various doses of EU-GMP certified *Cannabis sativa* L. No histological changes occurred regardless of the dose, H&E coloration.

Based on the histopathological screening of 22 organs, no significant histopathological abnormalities could be identified in the control group [0 mg/Kg] or in the experimental groups, regardless of the administrated dose (Figure 2). For all that, some slight changes (e.g., lymphoid agglomerations in the large intestine submucosa and uromodulin deposits in the kidney) were recorded in all the experimental groups, including the control group, most probably engendered by the inter-individual variability. Furthermore, no lesions were observed in the groups exposed to higher doses.

#### 2.1.3. LD50 Estimation

**Figure 3:**
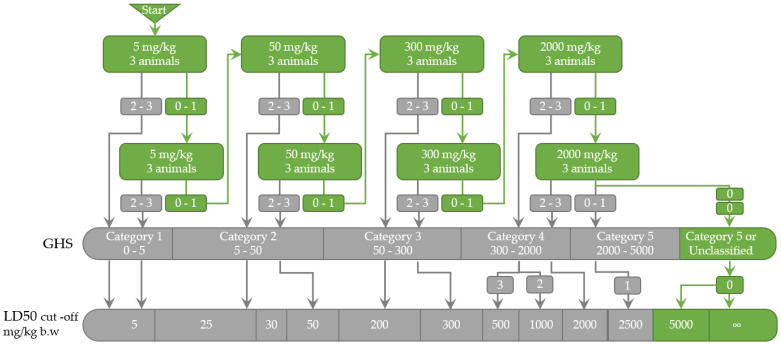
Overlay of the acute oral toxicity results over OECD 423 guidelines [40] on the *Cannabis sativa* L. administration. Per step three, animals of a single-sex (females) were used. 0, 1, 2, 3: number of moribund or dead animals at each step. GHS, Globally Harmonized Classification System for Chemical Substances and Mixtures (mg/kg b.w.); LD50, the median lethal dose (the dose of a test substance that is lethal for 50% of the animals in a dose group); b.w., body weight.

During the entire 14-day acute oral toxicity experiment, no deaths or significant organ physiological or functional alterations were recorded after the administration of the EU-GMP *Cannabis sativa* L. Therefore, based on OECD 423 methodology, the LD50 for oral administration in rats of Cannabixir^®^ Medium Flos was estimated to be equal to or higher than 5000 mg/kg (Figure 3). Furthermore, based on US FDA guidance [39], the estimated LD50 in rats of ≥5000 mg/kg was translated into a human equivalent dose for LD50 of 806.45 mg/kg. Accordingly, in order to reach potentially toxic levels, an adult human of 70 kg would have to ingest, in a short period of time, over 50 g of Cannabixir^®^ Medium Flos dried inflorescence, which by far exceeds any potential realistic use scenario in a legalized environment.

### 2.2. Pharmacokinetic Profile

#### LC-MS Analysis: Cannabinoids Concentration in Rat Serum

The extraction method of 11 natural cannabinoids from rat serum and a liquid chromatography-tandem mass spectrometry method was developed and validated in the present study based on previous research studies. Analyses carried out with the Agilent liquid chromatograph coupled to an electrospray triple-quadrupole 6410 allowed the identification and quantitation of 11 cannabinoids based on the optimization of mass spectrometer fragmentor and collision energy for the most abundant fragment ion. The LC-MS analysis for each sample was performed in 16 min, and the chemical compounds eluted from the separation column between 5.8 and 9.5 min. The method developed was applied for serum samples collected from rats to which doses of 5, 50, 300 and 600 mg/kg were administered and allowed the identification and quantitation in the samples of high amounts of THCA-A and CBGA and low amounts of CBVA, CBDA, CBCA, and Δ9-THC.

**Figure 4:**
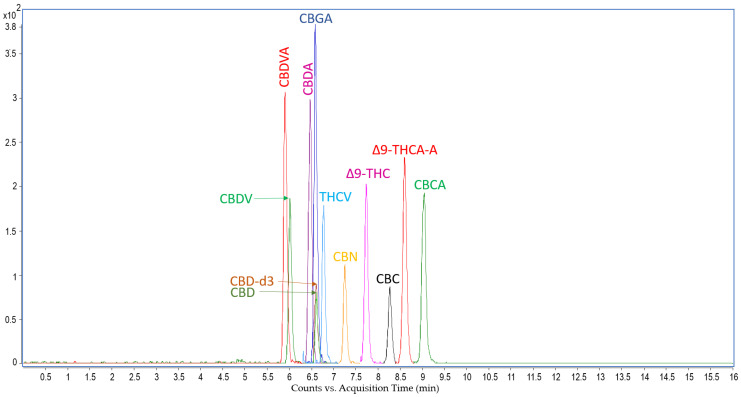
Overlaid mode representation of multiple reaction-monitoring chromatograms obtained by LC-MS/MS analysis of a sample resulted after extraction of cannabinoids from neat rat serum, which was fortified with 50 ng/mL natural cannabinoids. For each of the 12 peaks observed in the combined chromatogram an annotation containing the abbreviation of each natural cannabinoid spiked in rat serum is provided: CBDV, cannabidivarin; CBDVA, cannabidivarinic acid; CBDA, cannabidiolic acid; CBD, cannabidiol; CBD-d3, cannabidiol-d3; CBGA, cannabigerolic acid; THCV, tetrahydrocannabivarin; CBN, cannabinol; Δ9-THC, Δ9-tetrahydrocannabinol; CBC, cannabichromene; Δ9-THCA-A, Δ9-tetrahydrocannabinolic acid; CBCA, cannabichromenic acid.

**Figure 5:**
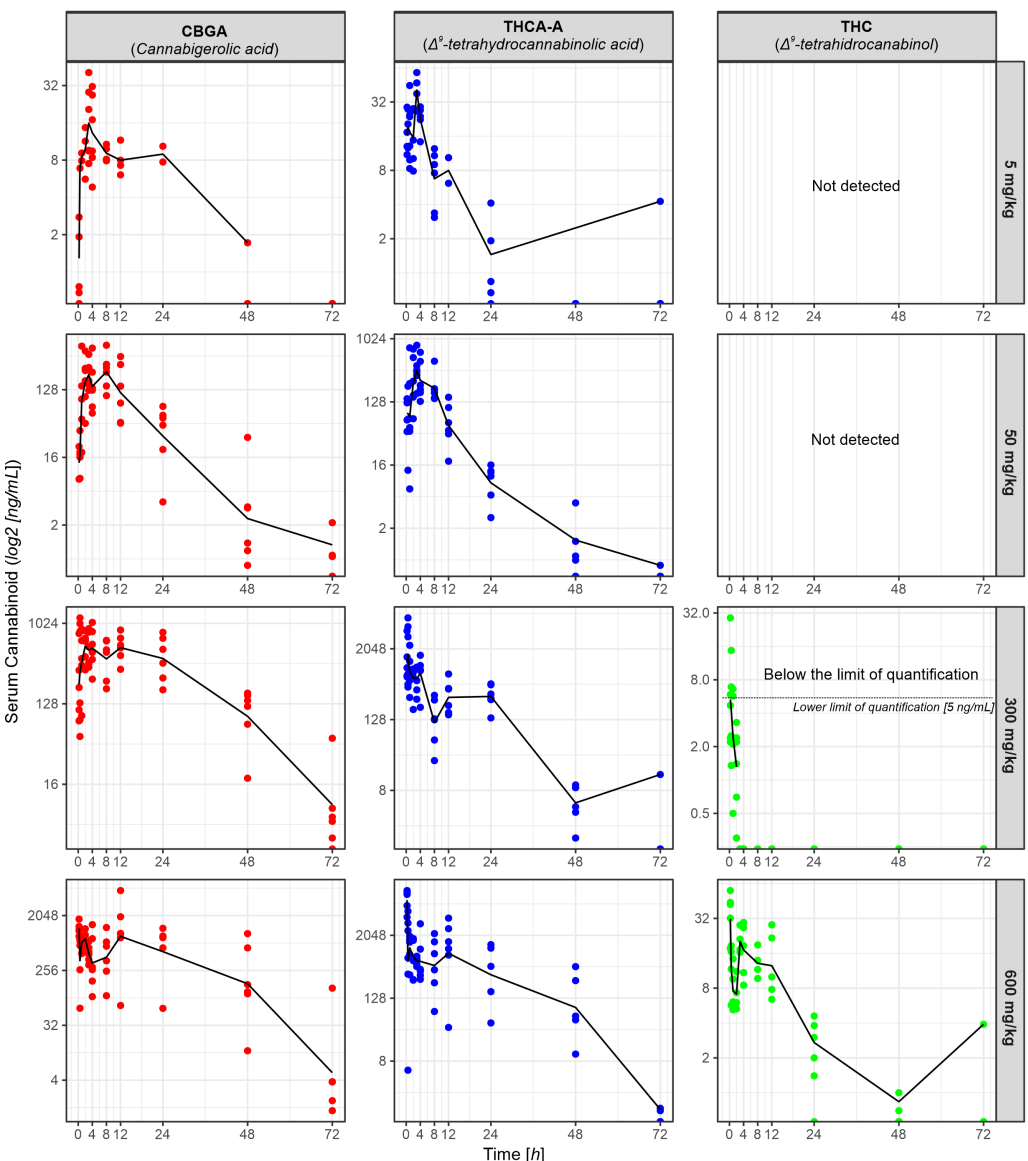
Cannabigerolic acid (CBGA), tetrahydrocannabinol acid (THCA-A) and Δ9-THC in rat serum for the 5 mg, 50 mg, 300 mg and 600 mg/kg dose groups.

Based on the characteristics of the Cannabixir^®^ Medium Flos phytocomplex, a non-compartmental pharmacokinetic analysis was performed. The analysis was performed using the WinNonlin software and calculated the following parameters: the maximum concentration (Cmax), the maximum time (Tmax), the observed area under the curve (AUC last), the total area under the curve (AVC inf), the half-time (T 1/2), the apparent distribution volume (Vd), and clearance (Cl; Table 2). The above parameters were calculated for both CBGA and THCA-A in rat serum for the 5 mg, 50 mg, 300 mg, and 600 mg/kg dose groups (Figure 4 and Figure 5).

## 3. Discussion

The aim of the present study was to evaluate the acute oral toxicity, LD50 and the pharmacokinetic profile of the major phytocannabinoids, after oral administration of a certified EU GMP batch of Cannabixir^®^ Medium Flos, in the form of dried inflorescence of EU-GMP certified *Cannabis sativa* L.

### 3.1. Acute Oral Toxicity

The oral administration route was used mainly due to the lack of a standardized procedure for inhalation in rats. In addition, there was no clear evidence that pointed out inhalation as the most suitable delivery route for medical purpose usage of various doses of EU-GMP certified *Cannabis sativa* L. Moreover, for humans, oral consumption has gained more and more ground, being considered by specialists to be much safer for the patient [41]. Despite the two centuries of cannabis use in Western medicine [42], there is limited information regarding the toxicity of oral administration or the pharmacokinetic profile of natural cannabinoids in both human subjects and laboratory animals [30]. Most of the toxicity studies were carried out using the main cannabinoids (THC, CBD) or their combinations, while the toxicity of the entire plant or its cannabinoid phytocomplex remains mainly unstudied. The latest acute toxicity reports are 50 years old, from the 1970′s, assessing the toxicity of THC and certain cannabis extracts [25,26,43,44,45].

In our study, the acute oral toxicity of EU-GMP-certified *Cannabis sativa* L. was evaluated. The first 24 h primary monitoring data showed writhing; moderate sedation; Straub phenomena; stereotypes; mild motor incoordination; short duration facial tremor, and abortive seizures during the initial 8 h, limited to 16% of the subjects for the 300 mg/kg group and loss of reactivity to touch during the first 8 h, for 16% of the subjects, for the 2000 mg. The health status did not exhibit any significant deviation from the normal. The general health parameters, such as mobility, general behavior, alertness state, and food or water intake, remained unaffected by oral administration of EU-GMP-certified *Cannabis sativa* L. The Straub phenomenon (1919), which necessitates the integrity of the lumbosacral medulla, is characterized by an S-shaped tail erection due to the dopamine release in the central nervous system [46]. Generally, convulsive responses in mice are evaluated by clonic maxillary movements and forepaws, immobility or Straub phenomena. Cannabigerol (CBG), cannabigerol acid (CBGA), cannabidivarinic acid (CBDVA), cannabichromevarinic acid (CBCVA) increase frequency of convulsions in Scn1A mice [47]. CBGA presents divergent effects on conventional models of seizures. These could be due to its effects on the transient receptor potential vanilloid 1 (TRPV1) receptors and gamma-aminobutyric acid (GABA) [48]. CBGA was pro-convulsive in doses of 10 and 30 mg/kg on seizure models with electric shocks [47]. Feigenbaum et al. [49] identified a non-psychotropic cannabinoid (HU-211) as being a functional blocker of the N-methyl-D-aspartate (NMDA) receptor, which might induce stereotypes, locomotor hyperactivity, tremor and seizures in mice. Also, cannabinoid type 2 receptors from the dopaminergic neurons might be involved in the modulation of stereotypes [50]. The neuromediators involved in the appearance of writhing are prostaglandins, pro-inflammatory cytokines like tumor necrosis factor-alpha (TNFa), interleukin 1 (IL1) and interleukin 8 (IL8). THCA-A is an inhibitor of TNF-alpha, cyclooxygenase 1 and 2 (COX1 and COX2), which might induce an antinociceptive action, which excludes it as a causal factor of writhing [51]. The actual occurrences might be allocated to artifacts, irritative phenomena not due to the administrations or hidden effects of the various molecules in the phytocomplex.

The gross necropsy could not observe any abnormalities in the body, internal cavities or organs. The biochemical analysis results show that the serum creatinine level has a small dose-dependent increase but without statistical significance compared to the control group. In addition, this does not correlate with the levels in urea, which show a downward trend, remaining below control group values, even for the 2000 mg/kg dose, which demonstrates normal or even improved kidney function. Furthermore, this was correlated with the morphological aspects revealed by the histopathological studies. Thus, at the renal level, hyaline areas were observed in the urinary tubules in the paracortical area, represented by uromodulin deposits, at all doses tested, except for the group that received the highest dose, where these changes were less pronounced. Recent studies [52,53,54,55] suggest that the presence of a high level of uromodulin (in urine or serum) is independently associated with a reduced risk of incident acute and chronic kidney disease, progression of kidney disease, and cardiovascular and mortality outcomes. In addition, serum levels of uromodulin are inversely correlated with markers of systemic inflammation [56,57]. From the point of view of biochemical parameters relevant to liver function, an inversely proportional distribution of AST values can be noted in relation to the administered doses, the average values in the batch of 2000 mg/kg being slightly below the reference limits for this parameter, but without statistical significance when compared to the control group. Also, the ALT values do not show significant deviations in relation to the administered doses, remaining close to the lower value of the reference interval. This fact is in contradiction with previous studies that incriminate the role of cannabinoids and, more precisely, the activation of CB1 and CB2 receptors, respectively, and of probably other yet unidentified receptors, in the pathogenic chain of hepatic steatosis and non-alcoholic fatty liver [58,59,60]. Enunciating and explaining these mechanisms goes beyond the scope of this article, but it should be mentioned that from a morphological point of view, in the present study, no specific pathological changes were detected that would raise the suspicion of liver damage. Moreover, ALP values, in conjunction with TBA, reveal a normal functionality of bile secretion and bile acid metabolism, with a slight increase in both parameters compared to the control group, but without statistical significance and without exceeding the reference values for either of the two parameters. This data is indirectly supported by the TP and ALB values, which are in the reference range and are thus relevant to the normal function of both hepatic synthesis and metabolism, as well as a normal function of renal filtration and purification, without raising suspicion of any organic injuries or functional anomalies. All this is also confirmed by the histological analysis, where no changes were detected. It is also worth mentioning the blood glucose levels, which are below those recorded in the control group, but without being below the safety threshold, a fact that can support the hypothesis formulated and documented in established studies on the benefits that cannabinoid compounds have on regulating the mechanisms of insulin resistance and the modulation of complex metabolic pathways, both central and peripheral on energy metabolism [61,62,63]. Thus, viewed as a whole, the changes in the previously mentioned biochemical markers, corroborated with the macroscopic and microscopic analysis of the target organs, do not raise the suspicion of a potential toxic effect of the tested compound at any of the doses administered during the study.

### 3.2. Pharmacokinetics

There are few studies that attempt to test acute toxicity by oral administration of cannabis plant powder [25,26,27,45]. It is known that the cannabis plant contains more than 500 different chemical compounds, out of which more than 100 are cannabinoids. Among these, the most represented are THC (delta 9-THC, delta 8-THC), cannabigerol (CBG), CBD, cannabinol (CBN), cannabidivarin (CBDV), and their acid precursors: THCA-A, cannabigerol acid (CBGA), CBDA, cannabinol acid (CBNA), and cannabidivarin acid (CBDV-A). Through successive reactions involving decarboxylation, auto oxidations, thermal degradation, and photolysis, a tight interdependency is created in the synthesis of these compounds (CBGA→THCA-A→THC→CNB, etc.) [64].

After inhalation administration, the THC plasma concentration vs. THCA-A is significantly higher when compared with the oral administration. This is explained by the fact that the acid precursor THCA-A, under the influence of temperature, decomposes to THC. An efficacy study demonstrated that the amount of THC from any standardized THCA-A is in agreement with the decarboxylation kinetics, pointing to the fact that contamination with THC is inevitable [48]. After the oral administration of the EU-GMP *Cannabis sativa* L. plant, higher plasma levels of the acid precursors were detected vs. the THC levels. These precursors can act through direct or indirect mechanisms on the cannabinoid receptors, can have their own actions or lack any pharmacological action, and also, they can reciprocally influence their own pharmacokinetics [65]. For such an ample phytocomplex, the most adequate preliminary analysis of the pharmacokinetic profile was a non-compartmental method. The parameters obtained in this way are useful for expanding the pharmacokinetic profile via compartmental analysis using the following models: mono-compartment, bi-compartment, and so on. Using supplemental equations on data from Table 2, from which we calculated the elimination constant (Ke) for the doses taken in the study, we observed a decrease in the elimination constant (Ke) proportional to the dose increase.

In a simulated calculation,

Cmax = (D × Fabs)/(Vd × Fep)

where Fabs is the absorbed fraction, Fex is the absorbed excretion fraction, and D is the absorbed dose administered; we observed that the Fabs/Fep ratio is supraunitary for a dose of 300 mg/kg, unitary for a dose of 50 mg/kg and subunitary for the dose of 5 mg/kg. This data obtained for large (supra unitary ratio, low Ke, high Vd, and a high T 1/2) suggest that it would be necessary to identify the adequate compartmental pharmacokinetic model in order to better shape a more comprehensive pharmacokinetic profile.

For the 300 mg/kg dose, the supraunitary ratio, low Ke, and high Vd could be attributed to the following: the presence of active metabolites, saturation of the metabolization systems, saturation of the transport systems (absorption-elimination), and the presence of cannabinoids that at doses of 5 and 50 mg were in too-low amounts to have biological actions; these parameters may be the result of the synergic or concomitant activities of the cannabinoids present in the phytocomplex. These cumulative tendencies we observed may explain some biological alterations we described in the evaluation of the acute toxicity (Section 2.1.1).

In the literature, THCA-A is a weaker agonist of CB1 and CB2 receptors than THC, with a higher affinity for CB1. THCA-A attenuated emesis and vomiting in rats by activating CB1, the effect not being due to the conversion to THC. THCA-A also seems to lack the psychotropic effects of THC. Because THCA-A has a low penetrability of the blood-brain barrier (BBB), it is conceivable that the actions are peripheral. This low penetrability of the THCA-A might be due to the carboxyl group, which reduces the lipid solubility of the molecule. THCA-A can effectively interact with the transient receptor potential melastatin-8 (TRPM8) channels, which are strongly involved in both acute and chronic pain modulation [66], can inhibit the release of TNF-alpha and stimulate and desensitize a series of other cation channels of the transient receptor potential (TRP) family. Inhibiting the enzymes degrading the endocannabinoids, THCA-A stimulates the increase of their levels. In vitro, in a model of Parkinson’s disease, THCA-A inhibits COX1 and COX2 and reduces the viability of various lines of cancerous cells as it significantly enriches the altered morphology of neurites, in a model of Parkinson’s disease [67].

THCA-A has also demonstrated a low binding for CB1 and CB2 receptors, at least 62 times smaller than that of THC for CB1 and 125 times smaller than THC for CB2. In efficacy tests, THCA-A slightly inhibited AMPc at CB1, which suggests a weak agonist activity and no efficacy for CB2 [68].

CBGA has antioxidant, antibacterial, neuromodulator and neuroprotective effects [69] while being an effective modulator of TRPV 1, TRPV 3 and TRPV4 channels [70]. Members of TRPV and TRPM subfamilies have high expression levels in neurons mediating neuropathic pain [71,72,73,74]. Interestingly, members of the TRPM families are expressed in the hippocampus and basal ganglia, brain structures that display a significant loss of neurons at the initial stages of the development of neurodegenerative diseases, such as Alzheimer’s disease. The specific role of TRPs in these types of disorders and related pain symptoms has not been studied in detail. However, several lines of evidence indicate a relationship between TRPs, pain and neurodegeneration, particularly related to inflammation [74]. CBGA also inhibits COXl and COX2, being a good candidate for the treatment of inflammatory pain [75]. The two compounds determined, CBGA and THCA-A, present varied and significant positive pharmacological effects while lacking the psychotropic effects of the THC alone.

Considering the reduced toxic effects observed for the dose sequences used in our study, and the calculated LD50, it is most likely that even the accidental ingestion of large doses of Cannabixir^®^ Medium Flos may be harmless to humans. At the same time, the possible favorable pharmacological effects, especially due to the high plasma levels of THCA-A and CBGA after oral administration, warrant a more in-depth exploration of this product in order to identify its potential therapeutic benefits.

The accurate extrapolation of animal data directly to humans may not be fully guaranteed due to interspecies variation in anatomy, physiology, and biochemistry, but they could aid in comprehending acute toxicity mechanisms and health hazards, thereby enhancing the generation of adverse outcome pathways. In addition, appropriate target engagement and safety studies should help define clinically meaningful doses and therapeutic windows. In future studies, we will perform safety screening and chronic toxicity of the phytocomplex. Studies to investigate the product’s efficacy in a broad range of diseases, as well as to establish an interval for the effective dose and a frequency of administration, are additional research directions that can be pursued following the current investigation.

## 4. Materials and Methods

### 4.1. Plant Material

A certified EU GMP batch of Cannabixir^®^ Medium Flos (PZN: 7001905), in the form of a dried inflorescence of *Cannabis sativa* L. with 15.6% THC and <1% CBD, was sourced from Cansativa GmbH (Mörfelden-Walldorf, Germany). The completely dried plant material was ground using an electrical mortar grinder RM 200 (Retsch GmbH, Haan, Germany), and the obtained powder was sieved through a 125 microns strainer (BSS Mesh No. 120).

### 4.2. Reagents

Formulation: carboxymethylcellulose sodium salt (Na-CMC) was purchased from Alfa Aesar GmbH & Co. (Kandel, Germany).

Biochemistry: all the reagents used for biochemical analysis were purchased as in vitro diagnostic ready-to-use reagents from PZ Cormay (Warsaw, Poland) and BioSystems S.A. (Barcelona, Spain) and used for the automatic analyzer ACCENT-200 (PZ Cormay, Warsaw, Poland) according to manufacturer’s instructions.

Histopathology: all the reagents used for tissue fixation, processing, embedding and staining were histological grade, purchased from Richard–Allan Scientific (Kalamazoo, MI, USA), and used according to manufacturer protocol.

LC-MS analysis: each natural cannabinoid used in the present study was purchased from Cayman Chemical in the form of a certified reference material supplied at a concentration of 1 mg/mL in either methanol or acetonitrile. Acetonitrile was purchased from VWR Chemicals (Rosny-sous-Bois, France) and was of HPLC, UHPLC and LC-MS grade. Methanol was purchased from LAB-SCAN Analytical Sciences (Gliwice, Poland) and was of HPLC grade. Formic acid 99.7% was from Merck (Darmstadt, Germany). Ultrapure water (0.055 μS/cm c) was produced by an Arium Mini Plus UV system (Sartorius Lab Instruments Gmbh & Co., Goettingen, Germany).

### 4.3. Animals

Two hundred ninety-four female Sprague–Dawley rats (8–12 weeks, 200–300 g) were housed in the CEMEX animal research facility of the Grigore T. Popa University of Medicine and Pharmacy, Iasi, Romania, in a controlled environment (20 ± 4 °C room temperature, 50 ± 5% relative humidity, and an artificial light-dark cycle of 12 h), using individually ventilated cages (IVCs), with ad libitum access to food and water, and acclimatized for at least 2 weeks before the experimental protocols. The experimental study was performed in accordance with the European Directive 2010/63/EU and has been approved by the university’s Research Ethics Committee (no. 47/17.02.2021) and authorized by the Romanian National Sanitary Veterinary and Food Safety Authority (no. 34/07.04.2021).

### 4.4. Acute Oral Toxicity Evaluation

The acute toxicity study was carried out following OECD 423: Guideline for testing of chemicals—Acute Oral Toxicity [40]. This method allows us to estimate the median lethal oral dose (LD50) and to classify the substance, plant or mixture of substances in the Globally Harmonized Classification System for Chemical Substances and Mixtures (GHS).

#### 4.4.1. LD50 Estimation

Due to the lack of recent and reliable information regarding the LD50 for *Cannabis sativa* L. or the possibility of making an informed decision on the dose with a mortality likelihood, we used the minimum starting dose level, 5 mg/kg, accordingly to the OECD 423 [40]. Therefore, the LD50 for oral administration of *Cannabis sativa* L. has been estimated using 4 dose levels, 5, 50, 300, and 2000 mg/kg. A Na-CMC 0.1% aqueous suspension was used as a vehicle to provide uniform doses of *Cannabis sativa* L. Nine groups have been defined, of 3 animals each, 2 groups for each dose level, and a group for the vehicle (Na-CMC 0.1%), to which the compound suspension was administrated intragastrically using a no. 16 rodent gavage needle.

#### 4.4.2. Monitoring and Measurement of Vital Signs

The primary monitoring protocol and parameters were adapted from the Primary Observation (Irwin) Test in Rodents [76], as we described below. After oral administration of *Cannabis sativa* L., each individual has been monitored continuously in the first 15 min and then regularly at 30 min, 2 h, 4 h, 8 h, and 24 h, respectively, using a video-tracking system. During the first 24 h, the primary monitoring has been focused on the detection of sedation, convulsions, motor incoordination, loss of balance, reactivity to touch, and tremors and/or stereotypes (head movements). Subsequently, general health parameters such as eating, locomotion, behavior, appearance, and weight loss were monitored and recorded daily for the next 13 days. During the entire monitoring period of 14 days, any abnormalities regarding the behavioral pattern or appearance (e.g., changes in skin and fur, eyes and mucous membranes, respiratory, circulatory, somatomotor activity, salivation, diarrhea, or lethargy) have been recorded.

#### 4.4.3. Paraclinical Evaluation: Gross Necropsy, Biochemistry, and Histopathology

Gross necropsy: on the 14th day after oral administration, all the animals were euthanized, and a cardiac puncture was performed to sample 2 mL of terminal blood in 2 3 mL clot activator vacutainer tubes for biochemistry assay. Postmortem, a complete necropsy was performed for all individuals, which consisted of a close examination of the external surface of the body, all the openings and the internal cavities (abdominal, thoracic and cranial cavity), as well as their contents. Subsequently, 22 internal organs (brain, spinal cord, eyes, stomach, small and large intestine, liver, kidney, adrenal glands, spleen, heart, thymus, thyroid, trachea and lungs, ovaries, uterus and cervix, vagina, bladder, lymph nodes, peripheral nerve, muscles, and skeletal bone with bone marrow) have been sampled and fixed in 10% formalin for a detailed histopathological examination.

Biochemistry assays: to investigate the toxic potential of orally administrated *Cannabis sativa* L. on primary organs responsible for drug metabolism (e.g., liver and kidney), several biochemical tests were conducted, as follows. After 30 min since harvesting, the vacutainer tubes were centrifuged at 1500× *g* for 15 min at 4 °C, and the separated serum samples were then subjected to biochemistry analysis using an ACCENT-200 Analyzer (PZ Cormay, Lomianki, Poland). The following parameters have been quantified through the serum biochemical analysis have been quantified the following parameters: glucose, total cholesterol, urea, creatinine, total protein, albumin, alanine transaminase (ALT), AST, alkaline phosphatase and bile acids.

Histopathology: all 22 internal organs harvested in 10% formalin after gross necropsy were let up to 48 h for fixation. Following the fixation step, each organ has been trimmed in organ-specific plans, according to the Revised guides for organ sampling and trimming in rats and mice—Parts 1 to 3 [77,78,79]. Two slices for each organ were then processed using the Excelsior™ AS Tissue Processor (Epredia Holdings Ltd., Portsmouth, NH, USA) and embedded in a single paraffin wax block. All embedded paraffin blocks were then sectioned using a semi-automatic microtome CUT 5062 (SLEE medical GmbH, Nieder-Olm, Germany) at 2 µm cutting thickness for kidneys and 3 µm for all the other organs. Two sections for each organ were then transferred to a microscope slide and stained using the hematoxylin and eosin (H&E) standard staining protocol. Subsequently, all the H&E-stained tissue microscope slides were examined by light microscopy using an Aperio AT2 DX slide scanner (Leica GmBh, Wetzlar, Germany) at 400× magnification scale. Photomicrographs were then analyzed and compared to the control by a veterinary histopathologist.

### 4.5. Pharmacokinetic Profile Evaluation

The pharmacokinetic profile of the EU-GMP-certified *Cannabis sativa* L. with 15.6% THC and <1% CBD was assessed using 4 dose levels, 5, 50, 300, and 600 mg/kg. Dose levels were chosen in accordance with OECD 417 guidelines being lower than 1000 mg/kg but still high enough to allow for the identification and measurement of natural cannabinoids in the serum [80]. Each dose level group consisted of 6 female Sprague-Dawley rats (8–12 weeks, 200–300 g) housed, cared and treated in the same conditions as the animals involved in the acute toxicity study. After gavage delivery of the compound, blood samples were collected, through a cardiac puncture, at 15 min, 30 min, 1 h, 2 h, 3 h, 4 h, 8 h, 12 h, 24 h, 48 h, and 72 h, respectively. The collected blood samples were then allowed to clot for 30 min at room temperature and centrifuged at 2000× *g* for 15 min at 4 °C. The separated serum samples were transferred immediately in microcentrifuge tubes and stored at −20 °C until THC and THCA quantification using LC-MS analysis were performed.

#### 4.5.1. Cannabinoids Analysis in Rat Serum

A method for simultaneous extraction and LC-MS/MS quantitation of 11 cannabinoids was developed and validated for the present study. Cannabinoid extraction from rat serum was performed according to the method described by Wakshlag et al. [81], with modifications. Rat serum (40 μL) was mixed with 10 μL water: methanol (1:1) and 10 μL internal standard (200 ng/mL of CBD-d3) diluted in water: methanol (1:1). Proteins were precipitated by adding 80 μL ice-cold acetonitrile to each sample followed by vortex 2 min at 6 °C, 1000 rpm and centrifugation at 4000 rpm for 10 min at 4 °C. The supernatant (100 μL) was mixed with ultrapure water (100 μL) in another 1.5 mL microcentrifuge tube and centrifuged again at 4000 rpm for 10 min at 4 °C. The supernatant (100 μL) was transferred to another 1.5 mL microcentrifuge tube and placed in ice until LCMS analysis. Ten μL of the sample were injected into a Phenomenex Kinetex C18 (100 × 4.6 mm, 2.6 μm, 100 Å) HPLC separation column connected in the column compartment of an Agilent 1200 HPLC coupled to an Agilent 6410 electrospray-triple quadrupole mass spectrometer [82]. A 10:1 post-column splitter was used. The column was equilibrated with mobile phase A (0.1% formic acid in water) and mobile phase B (0.1% formic acid in acetonitrile) at 50% B for 1 min. A linear gradient from 50% B to 90% B for 3 min was used and was held constant at 90% B for 6 min, then decreased to 50% B in 1 min and maintained at 50% B for 5 min. The flow rate was 0.8 mL, and the column temperature was 24 °C.

The mass spectrometer was operated in positive mode. The source parameters were a capillary voltage of 4000 V, a gas of temperature 325 °C, a gas flow of 8 L/min, and a nebulizer of 35 psi. The parameters of the mass spectrometer for each time and scan segment and the retention time for each compound are presented in Table 3.

#### 4.5.2. Instrumentation for Cannabinoids Extraction and LC-MS/MS Analysis

For the extraction of natural cannabinoids from rat serum, the following instruments were used: thermocycler for vortexing at temperatures above room temperature model Thermomixer F1.5 (Eppendorf, Hamburg, Germany), thermocycler for vortexing at temperatures below and above room temperature, model Thermal Mixer with a thermoblock for 24 places for 1.5 mL microtubes (Thermo Scientific, European Union), minicentrifuge model MSC-3000 (Riga, Latvia) equipped with R-1.5 rotor for 12 1.5 mL microtubes and benchtop centrifuge model SL 8R (Thermo Electron LED GmbH, Osterode am Harz, Germany) equipped with a rotor for 24 1.5 mL microtubes model Microclick 24 × 2. The LC-MS/MS system consisted of an Agilent 1200 liquid chromatograph containing a solvent compartment, a degasser (G1379B, Japan), a binary pump (G1312A, Waldbronn, Germany), an autosampler (G1329A, Waldbronn, Germany), a thermostatted column compartment (G1330B, Waldbronn, Germany), a 10:1 splitter (620-PO10, Analytical Scientific Instruments, Richmond, CA, USA) coupled to a 6410A2k triple quadrupole mass spectrometer (Agilent, Santa Clara, CA, USA) equipped with electrospray source and a Nitrogen generator N118LA, Peak Scientific Instruments Ltd. (Inchinnan, UK).

The qualitative analysis of the chromatograms and mass spectra was performed by Agilent MassHunter Workstation Software Qualitative Analysis v.B.01.02 and the quantitative analysis by Agilent MassHunter Workstation Software Quantitative Analysis v.B.01.03. Linear calibration curves were obtained for all 11 cannabinoids using the relative response (peak area of each cannabinoid/peak area of internal standard) and the relative concentration (concentration of each cannabinoid/concentration of internal standard). A linear 7-point calibration curve with 1/×2 weighing and a concentration range of 5–1000 ng/mL in rat serum was employed for the quantitation of the serum samples collected in the present study. The analysis of carryover was performed by LC/MS analysis of a blank sample extracted from normal rat serum after LC/MS analysis of a sample extracted from rat serum fortified with 1000 ng/mL natural cannabinoids. According to the results, carryover was absent at this concentration for all tested cannabinoids.

#### 4.5.3. Pharmacokinetic Analysis

The results obtained by LC-MS dosing of major compounds of the EU-GMP-certified *Cannabis sativa* L. in serum were used for the pharmacokinetic analysis using a non-compartmental model (Linear Up-Log Down method) for extravascular dosing. Pharmacokinetic parameters were estimated using WinNonlin pharmacokinetic software (Version 64, Certara L.P., Princeton, NJ, USA). When performing non-compartmental analysis, the area under the concentration-time curve (AUC) is calculated to determine the total drug exposure over a period of time. Together with the maximum concentration of the drug at a time (Cmax) at the Tmax time point, these parameters are used to evaluate the systemic exposure to the compounds contained by the plant. Also, the half-time (T1/2) and the clearance (Cl) of the compounds have been calculated.

## 5. Concluding Remarks

Our results indicate that the toxicity and safety profile of the EU-GMP certified *Cannabis sativa* L. (tested as Cannabixir^®^ Medium Flos), after acute oral administration in rats, is very good, with a determined oral LD50 value human equivalent oral dose ≈ 806.45 mg/kg. The minor-to-moderate effects of the phyto-complex on the behavioral manifestations in a small number of animals do not present particular importance in the overall pharmaco-toxicological picture. The biochemical markers changes corroborated with the macroscopic and microscopic analysis of the target organs are in agreement with earlier studies on cannabinoids reported in the literature and do not raise the suspicion of a potential toxic effect of the tested plant at any of the doses.

The favorable pharmacokinetic profile of the EU-GMP certified *Cannabis sativa* L. (mainly due to the high plasma levels of THC-A and CBGA after oral administration) deserves a deeper exploration through future efficacy and chronic use studies, which could support its therapeutic potential in specific targeted pathologies or indications.

In this regard, it would be worth testing selective drugs targeting TRPs and/or COX to manage neurodegeneration and treat associated symptoms such as pain and cognitive/motor dysfunction. The evidence suggests that the effectiveness of pharmacological agents regulating TRP channel activity or COX enzyme to treat neuropathological processes and pain deserves further research.

## Figures and Tables

**Table 1:** Serum biochemical parameters of female rats after oral administration. Values are means with their standard errors.

Dose/Parameter	0	5	50	300	2000
	mg/kg				
CRE [mg/dL]	0.5 ± 0.02	0.5 ± 0.04	0.6 ± 0.02	0.5 ± 0.01	0.6 ± 0.03
AST [U/L]	111 ± 9.15	138.1 ± 23.18	137.1 ± 0.07	111.8 ± 12.19	85.9 ± 10.86
ALT [U/L]	38.7 ± 3.13	41.6 ± 4.11	42.7 ± 2.65	37.5 ± 1.91	41.8 ± 4.17
TC [mg/dL]	98.8 ± 32.01	66.8 ± 17.48	87.7 ± 17.08	91.7 ± 21.51	113.8 ± 13.99
GLU [mg/dL]	98.2 ± 35.65	75.5 ± 27.07	68.3 ± 11.55	83.5 ± 7.50	93.3 ± 10.73
ALB [g/L]	44.2 ± 3.13	37.6 ± 6.91	44.6 ± 3.81	43.2 ± 2.99	44.7 ± 2.09
ALP [U/L]	86.8 ± 14.83	110.8 ± 7.89	138.7 ± 4.58	96.2 ± 7.07	125 ± 14.72
TP [g/L]	72.5 ± 4.49	59.7 ± 5.08	75.4 ± 3.69	69.4 ± 2.48	81.4 ± 1.04
UREA [mg/dL]	35.5 ± 5.14	25.1 ± 1.72	27.3 ± 5.79	26.3 ± 1.00	29.4 ± 3.24
TBA [µmol/L]	61 ± 26.56	46.7 ± 14.21	73.0 ± 11.51	27.8 ± 6.09	79.7 ± 32.86
N	3	6	6	6	6

Mean values were not significantly different from that of the control group [0 mg/kg] based on a paired two-sample *t*-test for means. CRE, creatinine; AST, aspartate aminotransferase; ALT, alanine aminotransferase; TC, total cholesterol; GLU, glucose; ALB, albumin; ALP, alkaline phosphatase; TP, total protein; UREA, urea; TBA, Total bile acids.

**Table 2:** Pharmacokinetic parameters using Linear Up-Log Down method of non-compartmental model for extravascular dosing, after oral administration of the *Cannabis sativa* L.

Compound	Cmax (ng/mL)	Tmax (h)	T1/2 (h)	AUC Last (h*ng/mL)	AUC Inf (h*ng/mL)	Vd (L/kg)	Cl (L/h/kg)
	5 mg/kg Cannabixir^®^ Medium Flos (0.78 mg Δ9-THCA-A + Δ9-THC)						
Δ9-THCA-A	42.425	3	5.3374	197.236	260.802	23.0298	2.099
Δ9-THC	0	0	0	0	0	0	0
	50 mg/kg Cannabixir^®^ Medium Flos (7.8 mg Δ9-THCA-A + Δ9-THC)						
Δ9-THCA-A	404.16	3	5.4189	3163.1937	3172.8488	19.2193	2.4583
Δ9-THC	0	0	0	0	0	0	0
	300 mg/kg Cannabixir^®^ Medium Flos (46.8 mg Δ9-THCA-A + Δ9-THC)						
Δ9-THCA-A	2787.617	0.5	10.291	13,323.57	13,544.792	51.298	3.4552
Δ9-THC	BQL	BQL	BQL	BQL	BQL	BQL	BQL
	600 mg/kg Cannabixir^®^ Medium Flos (93.6 mg Δ9-THCA-A + Δ9-THC)						
Δ9-THCA-A	10,563.4	0.25	10.988	40,371.48	43,261.689	34.298	2.1635
Δ9-THC	34.916	0.25	7.080	284.342	287.2334	3328.818	325.867

Cmax, maximal concentration; Tmax, time to a maximal concentration; T1⁄2, half-life; AUC last, the area under the curve from the time of dosing to the time of the last measurable (positive) concentration; AUC inf, the area under the curve extended to infinity; Vd, the volume of distribution based on the terminal phase; Cl, total body clearance for extravascular administration; NA, not available; BQL, below quantitation limit.

**Table 3:** List of natural cannabinoids employed with the method described above and precursor ion, product ion, mass spectrometer parameters and retention time for each cannabinoid.

Time Segment	Compound Name	Precursor Ion	Product Ion	Dwell Time (ms)	Fragmentor Voltage (V)	Collision Energy (V)	Retention Time (min)
1	Cannabidivarinic acid	331.2	191.1	100	135	30	5.93
1	Cannabidivarin	287.2	165.1	100	135	22	6.02
2	Cannabidiolic acid	359.2	219.1	40	135	30	6.48
2	Cannabidiol	315.2	193.2	40	135	20	6.63
2	Cannabidiol-d3	318.2	196.1	40	135	22	6.64
2	Canabigerolic acid	361.2	219.2	40	135	25	6.61
2	Tetrahydrocannabivarin	287.2	165.1	40	135	20	6.79
3	Cannabinol	311.2	223.1	200	135	20	7.28
4	Δ9-THC	315.2	193.2	200	135	20	7.76
5	Cannabichromene	315.2	193.2	200	135	17	8.3
6	Δ9-THCA-A	359.2	219.1	200	135	30	8.6
7	Cannabicromenic acid	359.2	219.1	200	135	25	9

Δ9-THCA-A, Δ9-tetrahydrocannabinolic acid; Δ9-THC, Δ9-tetrahydrocannabinol.

## Data Availability

The datasets used and/or analyzed during the current study are available from the corresponding author upon reasonable request.
